# Continuous Stitches Versus Simple Interrupted Stitches During Anterior Colporrhaphy: A Randomized Controlled Trial

**DOI:** 10.3390/jcm14020534

**Published:** 2025-01-16

**Authors:** Christine Bekos, Sören Lange, Marianne Koch, Wolfgang Umek, Greta Lisa Carlin, Florian Heinzl, Barbara Bodner-Adler

**Affiliations:** 1 Department for Obstetrics and Gynecology, Medical University of Vienna, 1090 Vienna, Austria; marianne.koch@meduniwien.ac.at (M.K.); wolfgang.umek@meduniwien.ac.at (W.U.); greta.carlin@meduniwien.ac.at (G.L.C.); florian.heinzl@meduniwien.ac.at (F.H.); barbara.bodner-adler@meduniwien.ac.at (B.B.-A.); 2 Department of Gynecology, University Hospital Zurich, 8091 Zürich, Switzerland; soeren.lange@usz.ch

**Keywords:** colporrhaphy, anterior repair, pelvic organ prolapse

## Abstract

**Background:** The anterior vaginal wall is frequently affected by prolapse, which is frequently treated with anterior colporrhaphy. However, this repair has a high recurrence rate, and no standardized approach exists. Our study aimed to compare two suture techniques concerning postoperative outcomes. **Methods:** This randomized, single-center trial involved patients with symptomatic pelvic organ prolapse, assigned to either continuous or interrupted stitches during anterior repair. The primary outcome was subjective symptom improvement, assessed by the German pelvic floor questionnaire 6–12 months post-surgery. Secondary outcomes included anatomical results and surgery-related adverse events per Clavien–Dindo classification (CDC). A total of 42 patients were analyzed to achieve 80% study power. **Results:** No significant differences were found in the pelvic floor scores between the two groups, but both groups showed significant improvements in prolapse and all other domains assessed by the questionnaire. None of the patients reported a recurrence of symptoms or required re-treatment during the follow-up visits. In patients with continuous stitches, significantly more CDC 2 and fewer CDC 1 events were noticed. The baseline prolapse stage, prolapse domain scores, age, and the stitching technique did not significantly influence the treatment success. **Conclusions:** We were able to demonstrate that both suture techniques are comparable and effective in improving subjective symptoms after anterior colporrhaphy, with no significant difference in outcomes between the two methods. The choice of stitching technique did not impact the recurrence of symptoms or the need for reoperation.

## 1. Introduction

Lifetime risk for pelvic organ prolapse (POP) has been reported to be 11–19% in women undergoing surgery for prolapse or incontinence [1]. The segment most impacted by prolapse is the anterior vaginal wall [2] and anterior colporrhaphy (AC), with native tissue repair being the most common surgical technique used for its repair. However, high rates of anatomical recurrence, ranging from 0 to 92%, are documented [3,4]. Several risk factors for the high recurrence rates have been reported in the literature, including variations in the surgical technique [5,6]. Regarding the plication method of sutures and the efficacy and safety of anterior colporrhaphy when using either continuous stitches or simple interrupted stitches, no comparative data exist so far.

Consequently, it is crucial to develop standardization of the operation technique and reporting. Halpern-Elenskaia et al. [7] highlighted this issue by reviewing 40 randomized controlled trials, noting the absence of detailed descriptions of AC and variations in each procedural step. Similarly, Fairclough et al. [8] reached comparable conclusions after examining the native tissue anterior repair methods used by 30 surgeons in the UK.

It is well known that the continuous suturing techniques for perineal closure to repair episiotomy and second-degree perineal tears following childbirth are associated with less short-term pain when compared to interrupted stitches [9,10]. In another randomized controlled trial, repairs of episiotomies and perineal tears using continuous suturing were completed more quickly and required less suture material compared to interrupted suturing, without leading to an increase in complications [11]. There were no differences in the incidence of pain.

In a retrospective study [12], double-layered anterior colporrhaphy was implemented to improve anatomical cure rates for prolapse, showing an 81.7% cure rate for cystocele and a 91.7% improvement in quality of life among patients. The study suggests that double-layered anterior colporrhaphy is a reproducible technique with high patient satisfaction, warranting further investigation through randomized controlled trials.

A study investigating the impact of different suture materials on prolapse recurrence after anterior colporrhaphy retrospectively found no significant difference in recurrence rates among rapid absorbable multifilament, rapid absorbable monofilament, and slowly absorbable monofilament sutures [13].

Identifying surgical factors that could influence the results of prolapse surgery is essential, particularly since there is limited scientific evidence available to guide the selection of suture techniques in colporrhaphy. This is the first RCT aimed to investigate the effects of using either continuous stitches or simple interrupted stitches in POP patients undergoing anterior vaginal prolapse repair (i.e., anterior colporrhaphy) in view of subjective symptom improvement.

## 2. Materials and Methods

### 2.1. Study Design

This trial is a single-center, randomized, single-blind prospective study designed to evaluate whether continuous sutures are superior to simple interrupted stitches, concerning the primary outcome in POP patients undergoing anterior colporrhaphy. Importantly, both the outcome assessors and the participants were blinded to the treatment assignments. Postoperative follow-up occurred between 6 and 12 months. All study patients underwent a standardized urogynecological examination, and prolapse was assessed and classified according to the ICS POP-Q assessment [14]. Regarding subjective outcomes, all study participants received the German version of the pelvic floor questionnaire [15]. This study was registered at clinicaltrials.org (NCT05449054).

This study was carried out at the urogynecological outpatient clinic within the Division of General Gynecology and Gynecologic Oncology, Department of Obstetrics and Gynecology, at the Medical University of Vienna.

### 2.2. Participants and Recruitment

The study population included women aged 18 and older who were referred to our urogynecological outpatient clinic for symptomatic POP. Eligible participants were those with anterior vaginal wall prolapse extending beyond the hymen (POP-Q point Ba > 0) with a central defect, experiencing vaginal bulge symptoms, and having an indication for re-constructive pelvic floor surgery, including anterior colporrhaphy with native tissue repair. Exclusion criteria contained recurrence of anterior vaginal wall prolapse, lateral defect, mesh use, or planned obliterative surgery. Additionally, patients with known or suspected pelvic malignancy, those currently undergoing systemic glucocorticoid treatment, and women who were unable or unwilling to participate were excluded from the trial.

### 2.3. Randomization

Randomization was performed by the randomizer of the Medical University of Vienna; subjects were stratified by parity (Primipara/Multipara) and randomized in blocks of four. To avoid revealing the specific procedures, the medical records documented the surgical procedure performed without specifying the type of suture used for anterior colporrhaphy. The study surgeon, rather than other research staff, collected intraoperative data.

### 2.4. Intervention

Anterior colporrhaphy was conducted in a traditional and standardized manner, adhering to our surgical policy. For cases of multicompartment prolapse, additional procedures (such as vaginal prolapse hysterectomy, sacrospinous ligament suspension, superficial perineorrhaphy, McCall’s culdoplasty, or posterior repair) were performed according to each surgeon’s preferred technique.

All surgeries were performed vaginally with native tissue repair under strict aseptic conditions, with patients in the dorsal lithotomy position. Preoperatively, the bladder was emptied using a thin disposable catheter, and antibiotic prophylaxis (cefazolin) was administered 30 min before incision. Hydrodissection, using a combination of adrenaline, saline, and local anesthetic, was applied, followed by a vertical anterior vaginal incision from the apex to two centimeters short of the external urethral meatus, using either electrosurgical instruments or a scalpel, based on the surgeon’s preference. The vaginal epithelium was grasped on both sides, and the fibromuscular layer of the anterior vaginal wall was sharply dissected laterally to the inferior pubic ramus. The bladder was fully dissected from the apex to 4–6 cm from the pubic ramus. Depending on preoperative randomization, patients received either continuous or interrupted stitches for plication during anterior colporrhaphy, using absorbable sutures (Vicryl 2/0) placed no more than 0.5 cm apart. Trimming of the vaginal skin was performed if necessary, and the anterior vaginal skin was closed using a continuous suture technique with 2/0 Vicryl sutures. The three participating surgeons were experienced in prolapse surgery and were part of the urogynecology core team.

At our institution, perioperative management is standardized, including preoperative single-dose antibiotics, a vaginal pack, and an indwelling urinary catheter for 24 h post-surgery. Postoperatively, all patients had their postvoid residual volume measured on the first day after surgery. A postvoid residual volume of 2x < 100 mL was considered normal, requiring no further follow-up. Volumes > 150 mL were deemed abnormal, necessitating clean intermittent self-catheterization until volumes consistently fell below 150 mL. Patients received standard analgesic therapy per the local hospital protocol (Metamizol 1 g intravenously, three times daily). Additionally, patients were advised to rest for two weeks post-operation and avoid strenuous physical activity. Follow-up visits were scheduled at 4–6 weeks, 6 months, and 1 year in our outpatient clinic, as per our study protocol. Urogynecological examinations were conducted at each visit using the POP-Q measurement system during maximum Valsalva effort in the seated semi-lithotomy position. Objective anatomical cure was defined as a Ba point < −1.

Recurrence was defined as POP-Q ≥ Stadium 2b combined with prolapse-associated symptoms.

Postoperative functional results for symptoms, quality of life, and sexuality were evaluated with the German version of the pelvic floor questionnaire (PFQ).

The PFQ is composed of four domains: bladder, bowel, pelvic organ prolapse, and sexual function. Each domain includes various questions that evaluate the severity and condition-specific quality of life. Questions are rated on a scale from zero to four. The total score for each domain is divided by the maximum possible score and then multiplied by ten, resulting in a value between zero (0 = no symptoms) and ten (10 = maximum symptoms) for each domain. Baessler et al. have published the results of the validation study and scoring system [15].

### 2.5. Primary and Secondary Outcome Measures

The primary outcome of interest was the improvement in subjective symptoms, as measured by the PFQ, evaluated 6–12 months post-surgery.

Secondary outcomes included the anatomical results for each compartment (POP-Q point, changes in POP-Q values from baseline), condition-specific quality of life (changes from baseline), and the rate of adverse events related to anterior colporrhaphy. Intraoperative adverse events included bladder injury, ureteral obstruction, and significant bleeding, while postoperative events encompassed hematoma development, vesicovaginal fistula, ureteral obstruction, urinary tract infection, incomplete bladder emptying, overactive bladder or stress incontinence symptoms, suture erosion, vaginal wound dehiscence, infection, or granulation tissue. Additionally, operation time, estimated blood loss, and the use of painkillers during the hospital stay were evaluated.

Postoperative complications were graded according to the Clavien–Dindo classification (CDC) [16].

### 2.6. Sample Size and Power Considerations

Alteration of the prolapse domain score obtained from the pelvic floor questionnaire before and after intervention (between baseline and 12 months follow-up) was compared between patients receiving continuous stitches versus simple interrupted stitches for anterior colporrhaphy. Schoenfeld at al. [17] observed a mean prolapse domain score of 3.33 with a standard deviation of 2.2; we assumed that the standard deviation of the post-interventional treatment would be smaller. Already with a modest drop to 2 with roughly 18 patients per group, we would be able to detect a difference of 2 between the post-intervention values of the two groups with a power of 80% when using a *t*-test. Considering that the Pitman asymptotic relative efficiency of the Wilcoxon rank sum test (which is just a special case of ordinal logistic regression) with respect to the *t*-test equals 0.864, we calculated a sample size needed of 21 patients per group for our multivariate analysis using the same assumptions. The number of predictors in this approach was then justified by the (rather conservative) rule of ten. Taking into account an expected dropout rate of 20%, 26 patients were included per group.

### 2.7. Data Analysis

Numerical data are represented by median and interquartile range, while categorical data are given by absolute and relative frequencies.

Groups were compared using the Wilcoxon rank sum test (numerical data) as well Barnard’s test and χ2-test (categorical data, depending on the degrees of freedom). Additionally, multivariate analysis was conducted (ordinal logistic regression). We considered *p*-values below 0.05 as statistically significant.

The following statistical software was used: R version 4.4.1 [18] and packages tidyverse v2.0.0 [19], viridis v0.6.5 [20], flextable v0.9.6 [21], officer v0.6.6 [22], and Barnard v1.8 [23].

## 3. Results

**Figure 1:**
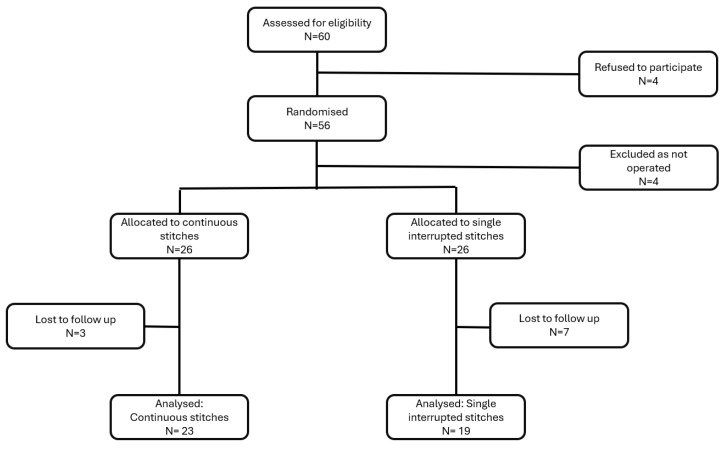
Flow diagram of patient distribution.

Fifty-six patients were enrolled and randomly assigned in this trial from July 2021 to October 2022. The allocation and follow-up are displayed in Figure 1.

Demographic and preoperative anatomic data were similar between groups (Table 1).

There was no difference between groups in type and number of concomitant procedures performed (Table 1). Twelve women (28.6%) underwent hysterectomy with McCall’s culdoplasty, and 30 patients (71.4%) underwent sacrospinous suspension at the time of anterior colporrhaphy.

No significant difference was noted in operating time (Table 2). There were no cases of ureteral, bladder, or bowel injury during operation. In general, the postoperative complication rate was low. However, we observed some differences between the groups (*p* = 0.023). In the continuous stitches group, significantly more CDC 2 and significantly fewer CDC 1 events could be observed. In the continuous stitches group, more urinary tract infections were observed but less stress incontinence and local infection/granulation tissue was involved.

However, all complications were resolved by the time of discharge, and there were no reported complications during the follow-up examinations.

At the follow-up visit, 26 patients had stage 2 prolapse, and 5 patients had stage 3 prolapse. None of our patients were reported to have a recurrence of symptoms. None of our patients were reoperated during the follow-up period.

**Figure 2:**
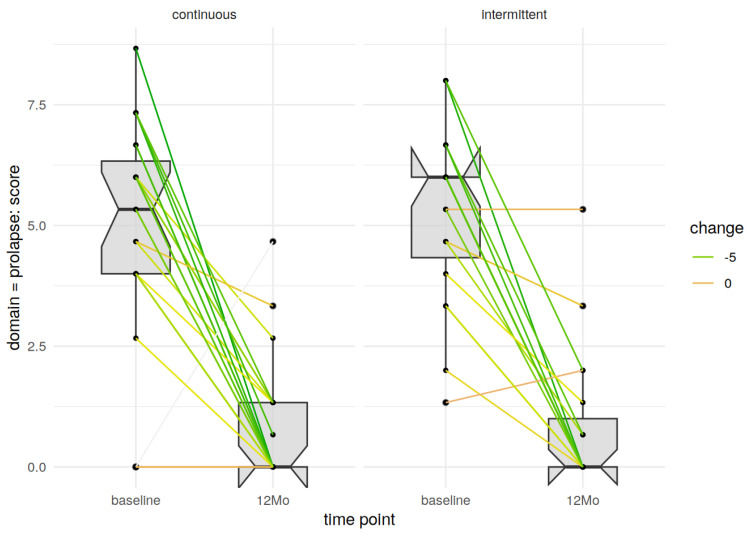
Prolapse domain of the PFQ at baseline and after 6–12 months in the continuous stitches and interrupted stitches group.

**Figure 3:**
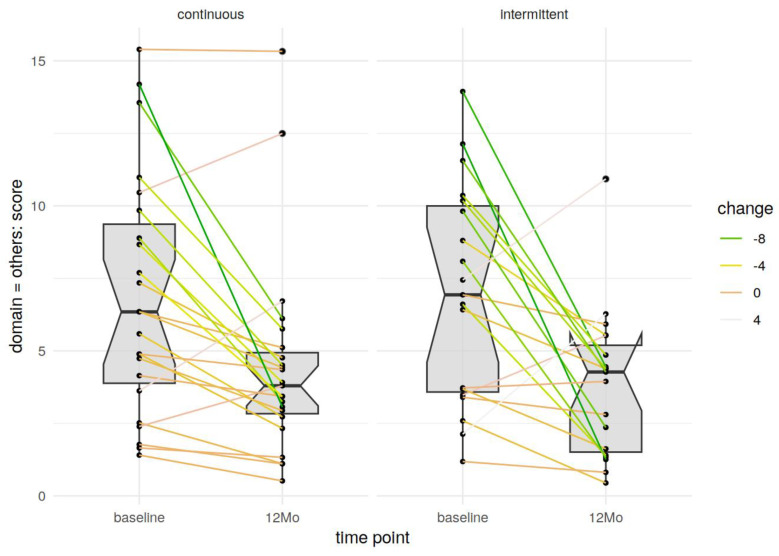
All other domains of the PFQ at baseline and after 6–12 months in the continuous stitches and interrupted stitches group.

There were no significant differences in any of the scales or subscales between groups. Both groups had significant improvements in the prolapse, but also all other domains assessed in the German pelvic floor questionnaire (Figure 2 and Figure 3).

The baseline prolapse stage, baseline prolapse domain score of the German pelvic floor questionnaire, age, and continuous stitching technique did not significantly influence the success rates of the treatment groups (Table 3).

The baseline prolapse stage, baseline all other domains of the PFQ, age, and continuous stitching technique did not significantly influence the success rates of the treatment groups (Table 4).

## 4. Discussion

These are the first results that showed that single interrupted stitches versus continuous stitches for an anterior colporrhaphy resulted in similar anatomical and functional outcomes 12 months post-surgery. Both suture techniques resulted in significant improvement in prolapse symptoms as well as in all other pelvic floor dysfunction symptoms.

In our study, the German pelvic floor questionnaire was used to assess the subjective symptoms regarding bladder function, bowel function, pelvic support, and sexual function. The median scores for these domains indicate relatively good outcomes postoperatively regarding overall pelvic floor function and quality of life. The preoperative scores were comparable to those suffering from POP reported by Schoenfeld M et al., 2013 [17].

Anatomical recurrence of POP (defined as POP-Q stage 3) could be observed in five patients, whereas four/five received continuous and one interrupted stitches. The majority of our patients (n = 26/42) were diagnosed with a POP-Q stage 2 at a follow-up of 6–12 months. This is even more than reported in the current literature, which describes a recurrence rate of 45.1% after traditional anterior vaginal repair [24]. However, the authors have to emphasize that none of our patients reported having subjective symptoms of recurrence, and none of our patients underwent retreatment (operation or pessary).

A prior article concluded that defining success after POP surgery should encompass symptomatic and anatomical criteria, the absence of retreatment, and consider the hymen as a benchmark for anatomical success [25]. Additionally, POP-Q stage 2 prolapse without symptoms is common and is not regarded as an indication for treatment [26].

Furthermore, our data revealed significantly more CDC 2 events in the continuous stitches group than in the interrupted stitches group. The higher complication rate can be explained by a higher number of UTIs observed in this group. Nine out of twenty-three (39.1%) patients in the continuous stitches group suffered from UTIs postoperatively, whereas they affected only one out of nineteen (5.3%) in the single-interrupted stitches group. We have no plausible explanation for this difference; however, we assume that the observed difference is likely due to chance. Further data would be necessary to make a definitive statement regarding this complication. However, all complications had resolved by the time of discharge, and there were no reported complications during the follow-up examinations.

### Limitations and Strengths of the Study

Notable strengths of our study are that all patients were operated by high-volume urogynecological surgeons in one single institution and therefore all patients were treated under the same conditions.

One limitation of the study is the high loss in the follow-up rate with 25% of the randomized patients. This high number is considered acceptable given the pandemic circumstances and the high age of some of our patients. Nonetheless, the lost patients might alter the results noticeably.

Another limitation of our study is that prolapse recurrence is not only influenced by surgical strategies, but also by other factors such as the width of the genital hiatus, levator avulsion, increased POP-Q stage, and a history of previous prolapse surgeries [27], rather than solely by the suture techniques employed. This highlights the multifactorial nature of the problem, where multiple elements contribute to the success of surgical interventions. All these factors could be possible confounders influencing our results.

Additionally, our study primarily addresses the comparison of suture techniques, but does not thoroughly address biomechanical or strategic factors—such as fascia repairs, ligamentous support, or use of mesh—known to have a more substantial impact on long-term recurrence risk than suture pattern alone. As a result, any suggestion of equal efficacy in preventing prolapse recurrence appears overly optimistic and not fully grounded in the multifactorial nature of the condition.

A 12-month follow-up may not fully capture the long-term effects and potential complications that can arise after surgical interventions. This could limit the understanding of the durability of surgical outcomes over a more extended period. While the 12-month follow-up provides useful early insights, the conclusions about recurrence prevention should be interpreted with caution. Further studies with extended follow-up are needed to draw more definitive conclusions regarding long-term results. A re-evaluation of outcomes at the 5-year mark would provide invaluable insights for both patients and healthcare providers.

## 5. Conclusions

In conclusion, our study suggests that both single interrupted and continuous stitches for anterior colporrhaphy yield similar short-term anatomic and functional outcomes, with significant improvements in prolapse and other pelvic floor symptoms 12 months post-surgery. However, the 12-month follow-up may not fully capture long-term outcomes or recurrence, and the study did not address other factors that may influence recurrence risk, such as biomechanical aspects or the use of mesh. Given these limitations, further research with extended follow-up is necessary to draw definitive conclusions about the long-term efficacy of these techniques. Future studies should aim to standardize surgical techniques and consider additional factors that contribute to long-term success.

## Figures and Tables

**Table 1:** Demographic and preoperative anatomic data.

		All	Interrupted Single Stitches	Continuous Stitches	*p* Value
Variable		42	19	23	
Age	median (IQR)	62.5 (54–71)	63 (52–71.5)	62 (56–70.5)	0.9496
	[95% CI]	[58–66]	[52–72]	[58–70]	
	NA	0 (0%)	0 (0%)	0 (0%)	
Parity	median (IQR)	2 (2–3)	2 (2–3)	2 (1.5–2)	0.3201
	[95% CI]	[2–2]	[2–3]	[2–2]	
	NA	0 (0%)	0 (0%)	0 (0%)	
BMI	median (IQR)	25.3 (23.13–26.82)	25.4 (22.55–26.6)	25.2 (23.36–27.05)	0.6766
	[95% CI]	[23.8–26.3]	[22–26.9]	[23.4–26.6]	
	NA	0 (0%)	0 (0%)	0 (0%)	
Menopausal Status	premenopausal median (IQR)	8 (19.05%)	6 (31.58%)	2 (8.7%)	0.0445
	perimenopausal median (IQR)	30 (71.43%)	13 (68.42%)	17 (73.91%)	
	postmenopausal median (IQR)	4 (9.52%)	0 (0%)	4 (17.39%)	
	NA	0 (0%)	0 (0%)	0 (0%)	
HRT	No	28 (66.67%)	10 (52.63%)	18 (78.26%)	0.0977
	Yes	14 (33.33%)	9 (47.37%)	5 (21.74%)	
	NA	0 (0%)	0 (0%)	0 (0%)	
Smoking	No	35 (83.33%)	14 (73.68%)	21 (91.3%)	0.1510
	Yes	7 (16.67%)	5 (26.32%)	2 (8.7%)	
	NA	0 (0%)	0 (0%)	0 (0%)	
Previous hysterectomy	No	40 (95.24%)	19 (100%)	21 (91.3%)	0.2181
	Yes	2 (4.76%)	0 (0%)	2 (8.7%)	
	NA	0 (0%)	0 (0%)	0 (0%)	
Previous incontinence operation	No	20 (47.62%)	9 (47.37%)	11 (47.83%)	0.9943
	Yes	22 (52.38%)	10 (52.63%)	12 (52.17%)	
	NA	0 (0%)	0 (0%)	0 (0%)	
Comorbidities	No	38 (90.48%)	16 (84.21%)	22 (95.65%)	0.2557
	Yes	4 (9.52%)	3 (15.79%)	1 (4.35%)	
	NA	0 (0%)	0 (0%)	0 (0%)	
PFQ all domains but prolapse	median (IQR)	6.51 (3.64–9.84)	6.93 (3.58–10)	6.34 (3.88–9.37)	0.8201
	[95% CI]	[4.74–8.67]	[3.48–10.18]	[4.15–8.89]	
	NA	0 (0%)	0 (0%)	0 (0%)	
PFQ bladder	median (IQR)	2.67 (0.94–4.89)	2.67 (1.78–3.89)	2.67 (0.89–4.89)	10.000
	[95% CI]	[2–4]	[1.56–4]	[0.89–4.89]	
	NA	0 (0%)	0 (0%)	0 (0%)	
PFQ bowel	median (IQR)	2.21 (0.96–2.87)	2.35 (1.03–4.12)	1.76 (1.03–2.5)	0.3281
	[95% CI]	[1.18–2.65]	[0.88–4.71]	[1.18–2.35]	
	NA	0 (0%)	0 (0%)	0 (0%)	
PFQ prolapse	median (IQR)	5.67 (4–6)	6 (4.33–6)	5.33 (4–6.33)	0.8380
	[95% CI]	[4.67–6]	[4–6]	[4–6]	
	NA	0 (0%)	0 (0%)	0 (0%)	
PFQ sex	median (IQR)	1.43 (0–2.38)	0.95 (0–2.14)	1.43 (0–2.62)	0.6406
	[95% CI]	[0–2.38]	[0–2.38]	[0–2.38]	
	NA	0 (0%)	0 (0%)	0 (0%)	
POP-Q stage	median (IQR)	3 (2.25–3)	3 (2–3)	3 (3–3)	0.0366
	[95% CI]	[3–3]	[2–3]	[3–3]	
	NA	0 (0%)	0 (0%)	0 (0%)	

IQR = interquartile range; CI = confidence interval; NA = not available; BMI = body mass index, HRT = hormonal replacement therapy; PFQ = pelvic floor questionnaire; POP-Q = pelvic organ prolapse quantification system.

**Table 2:** Comparison of results between the groups.

		All	Interrupted Single Stitches	Continuous Stitches	*p* Value
Variable		42	19	23	
Operation time	median (IQR)	73.5 (60.25–85.75)	75 (61.5–87.5)	73 (59.5–84.5)	0.7234
	[95% CI]	[68–77]	[61–95]	[60–82]	
	NA	0 (0%)	0 (0%)	0 (0%)	
Uterine sparing surgery	no	12 (28.57%)	5 (26.32%)	7 (30.43%)	0.8202
	yes	30 (71.43%)	14 (73.68%)	16 (69.57%)	
	NA	0 (0%)	0 (0%)	0 (0%)	
CDC	0	20 (47.62%)	10 (52.63%)	10 (43.48%)	0.0230
	1	12 (28.57%)	8 (42.11%)	4 (17.39%)	
	2	10 (23.81%)	1 (5.26%)	9 (39.13%)	
	NA	0 (0%)	0 (0%)	0 (0%)	
Adverse events	Bleeding	6 (24%)	3 (27.27%)	3 (21.43%)	0.0150
	Incomplete bladder emptying	4 (16%)	3 (27.27%)	1 (7.14%)	
	Infection/granulation tissue	2 (8%)	2 (18.18%)	0 (0%)	
	Stress incontinence	2 (8%)	2 (18.18%)	0 (0%)	
	UTI	10 (40%)	1 (9.09%)	9 (64.29%)	
	Vaginal wound dehiscence	1 (4%)	0 (0%)	1 (7.14%)	
	NA	17 (40.48%)	8 (42.11%)	9 (39.13%)	
postOP PFQ all domains but prolapse	median (IQR)	3.93 (2.33–5.05)	4.27 (1.51–5.19)	3.8 (2.83–4.94)	0.8201
	[95% CI]	[2.94–4.45]	[1.4–5.53]	[2.94–4.76]	
	NA	0 (0%)	0 (0%)	0 (0%)	
postOP PFQ bladder	median (IQR)	1.56 (0.67–2.33)	1.33 (0.56–2)	1.78 (1.33–2.44)	0.3224
	[95% CI]	[1.33–2]	[0.44–2]	[1.56–2.44]	
	NA	0 (0%)	0 (0%)	0 (0%)	
postOP PFQ bowel	median (IQR)	1.47 (0.59–2.35)	1.47 (0.59–2.35)	1.47 (0.74–2.21)	0.6847
	[95% CI]	[1.18–2.06]	[0.59–2.35]	[0.88–2.06]	
	NA	0 (0%)	0 (0%)	0 (0%)	
postOP PFQ prolaps	median (IQR)	0 (0–1.33)	0 (0–1)	0 (0–1.33)	0.9307
	[95% CI]	[0–0.67]	[0–1.33]	[0–1.33]	
	NA	0 (0%)	0 (0%)	0 (0%)	
postOP PFQ sex	median (IQR)	0 (0–0.48)	0 (0–0.24)	0.48 (0–0.95)	0.0741
	[95% CI]	[0–0.48]	[0–0.48]	[0–0.95]	
	NA	0 (0%)	0 (0%)	0 (0%)	
postOP POP-Q stage	median (IQR)	2 (1.25–2)	2 (1–2)	2 (2–2)	0.2570
	[95% CI]	[2–2]	[1–2]	[2–2]	
	NA	0 (0%)	0 (0%)	0 (0%)	
Difference in postOP PFQ all domains but prolapse	median (IQR)	−2.05 (−5.31–−0.55)	−2.14 (−6.27–−0.48)	−1.8 (−4.51–−0.6)	0.4950
	[95% CI]	[−4.26–−0.89]	[−6.76–−0.37]	[−4.26–−0.67]	
	NA	0 (0%)	0 (0%)	0 (0%)	
Difference in postOP PFQ prolapse	median (IQR)	−5 (−6–−2.83)	−6 (−6–−3)	−4.67 (−6–−3)	0.8986
	[95% CI]	[−6–−3.33]	[−6–−2.67]	[−6–−3.33]	
	NA	0 (0%)	0 (0%)	0 (0%)	
Difference in postOP POP-Q stage	median (IQR)	−1 (−1–0)	−1 (−2–0)	−1 (−1–−0.5)	0.9030
	[95% CI]	[−1–−1]	[−2–0]	[−1–−1]	
	NA	0 (0%)	0 (0%)	0 (0%)	

IQR = interquartile range; CI = confidence interval; NA = not available; CDC = Clavien–Dindo classification, PFQ = pelvic floor questionnaire; POP-Q = pelvic organ prolapse quantification system.

**Table 3:** Logistic regression for factors associated with success rates.

Predictor	Estimate	CI Low	CI High	*p*-Value
Continuous stitches	0.133	−1.182	1.496	0.845
Age	−0.027	−0.079	0.025	0.312
Baseline POP-Q stage	−0.235	−1.785	1.374	0.768
Baseline pelvic floor questionnaire prolapse domain	−0.086	−0.395	0.228	0.583

**Table 4:** Logistic regression for factors associated with success rates.

Predictor	Estimate	CI Low	CI High	*p*-Value
Continuous stitches	0.694	−0.540	1.976	0.286
Age	0.037	−0.010	0.088	0.143
Baseline POP-Q stage	−0.880	−2.316	0.495	0.226
Baseline pelvic floor questionnaire all other domains but prolapse	0.262	0.096	0.442	0.006

## Data Availability

Datasets are available on request from the authors.
